# Trends in cesarean delivery rates in primipara and the associated factors

**DOI:** 10.1186/s12884-020-03398-6

**Published:** 2020-11-23

**Authors:** Guoqiang Sun, Ying Lin, Honglian Lu, Wenjing He, Ruyan Li, Lijun Yang, Xian Liu, Hongyan Wang, Xuewen Yang, Yao Cheng

**Affiliations:** Obstetrics Department, Maternal and Child Health Hospital of Hubei Province, Tongji Medical College, Huazhong University of Science and Technology, Wuluo Road 745#, Hongshan District, Wuhan, 430070 Hubei China

**Keywords:** Primipara, Cesarean delivery, Annual percentage change, Average annual percentage changes, Child policy

## Abstract

**Background:**

Few studies have focused on cesarean delivery (CD) trends among primipara under the one-child and the two-child policies. This study aimed to explore the trends in CD rates among primipara during 1995–2019 and the associated factors with CD risk.

**Methods:**

This study obtained clinical data on primiparous mothers and newborns from 1995 to 2019 at a large tertiary hospital in Wuhan, China. Trends in CD rates were calculated using the joinpoint regression analysis. The Chi-square tests and log-binomial regression analyses were used to evaluate the associations between primary variables and CD risk.

**Results:**

CD rates showed a significant upward trend with an average annual percentage change (APC) of 2.2% (95% CI: 0.6, 3.8%) during the study period. In 1995–2006, the CD rates continued to increase with an APC of 7.8% (95% CI: 4.8, 10.9%). After 2006, the CD rates started to decline by an APC of − 4.1% (95% CI: − 5.5, − 2.6%). The CD rates non-significantly increased from 36.2% in 2016 to 43.2% in 2019. Moreover, the highest CD rate during 2015–2019 was observed on August 30 (59.2%) and the lowest on September 1 (29.7%). Primipara of older age and those with >3pregnancies had higher risks of CD. Furthermore, primipara who gave birth to newborns with low birth weight and macrosomia had higher risks of CD.

**Conclusions:**

Maternal and fetal as well as social and cultural factors may contribute to the rising trend of CD rates. Effective measures should be taken to control CD under the two-child policy, especially for primipara.

**Supplementary Information:**

The online version contains supplementary material available at 10.1186/s12884-020-03398-6.

## Background

Cesarean section is an effective way to improve the survival of mothers and newborns. However, the rapid increase in cesarean delivery (CD) adoption has not completely led a corresponding decrease in maternal and neonatal mortality. It has been suggested that the ideal CD rate to reduce maternal and neonatal mortality is approximately 19% [1]. In the past 25 years, the rising trend in CD rates has been observed worldwide. Recently, an increase in CD rates from 6.4% in 2010 to 14.4% in 2016 was observed in low- and middle-income countries [2]. It has been reported that the CD rate in China was 36.7% in 2018, one of the highest in the world [3].

Since the implementation of the family planning one-child policy in the 1980s, families having an ‘only-child’ have increased in number in the mainland China [4]. The two-child policy was proposed since 2007 and officially implemented in 2013. With the full liberalisation of the two-child policy at the end of 2015, the proportion of elderly multiparous women with a previous CD largely increased [5]. CD could result in poor elasticity of the uterus, which might lead to uterine rupture and postpartum hemorrhage. Most pregnant mothers with a scarred uterus are obliged to undergo a secondary CD. In addition, multiparous women with advanced maternal age and chronic diseases during pregnancy might also have a higher risk of CD [6]. It is difficult to reduce the CD rate among multipara in a short time. Therefore, in strategies for CD control, the focus should be on primipara. In China, the preference for CD among pregnant women or their families could have a large effect on the adoption of CD [7]. Maternal CD preference might be affected by traditional customs of a lucking day, children’s education, or other social factors [8]. Moreover, cultural factors are changing along with social development. Therefore, the trends in CD rates during such social transitions could not only highlight the progress and challenges in CD control, but also reflect the influence of social development on CD to a certain extent. This study aimed to explore the trends in CD rates among primipara during 1995–2019 in China and the factors associated with CD risk.

## Methods

### Data sources and study population

The participants included in the study were women who delivered their babies at the Maternal and Child Health Care Hospital of Hubei Province, which is the largest tertiary maternity and child healthcare centre in Hubei Province. In 2018, 22.6% of newborns in Wuhan were delivered at this hospital [9]. This retrospective study obtained clinical data of primiparous mothers (age at birth ≥16 years and gestational age ≥ 28 weeks) and newborns (fetal weight ≥ 1000 g) from January 1, 1995 to December 31, 2019. GDP per capita at childbirth year was obtained from the Wuhan Provincial Bureau of Statistics [10]. This study was approved by the Ethics Committee of Maternal and Child Health Hospital of Hubei Province.

### Variable definitions

Our dependent variable was the mode of delivery (vaginal delivery [VD] vs. CD). The independent variables related to maternal characteristics included the year of delivery; season of delivery (spring [March to May], summer [June to August], autumn [September to November], and winter [December to February]); maternal age at delivery (< 20, 20–24, 25–29, 30–34, and > 34 years); gestational age at delivery (pre-term delivery [< 37 weeks], term delivery [37–41 weeks], and post-term delivery [> 41 weeks]); gravidity (1, 2–3, and > 3); fetus number (singleton and multiparous); and maternal complications [11]. Additionally, the independent variables related to neonatal characteristics included fetal position (head, breech, other, and unclassified); fetal sex (male, female, and unclassified); and weight at birth (< 1500 g, 1500–2499 g, 2500–3999 g, 4000–4499 g, and > 4499 g).

### Data analysis

Chi-square tests were used to evaluate the differences in the primary variables including maternal and neonatal characteristics between VD and CD. Multiple logistic regression analyses were used to evaluate the effects of maternal and neonatal factors on the type of delivery. Potential confounding variables, including gestational week, complications, fetal position, and GDP per capita at childbirth year, were adjusted for. Results of multiple log-binomial regression analyses are reported as adjusted odds ratios (ORs) and corresponding 95% confidence intervals (CIs). All tests were two-sided, and *p* values < 0.05 were considered statistically significant. Statistical analyses were performed using SAS 9.4 for Windows.

Trends in CD rates were calculated using the joinpoint regression analysis proposed by Kim et al. (Joinpoint Regression Software, Version 4.0.4–May 2013; Statistical Methodology and Applications Branch, Surveillance Research Program of the US National Cancer Institute) [12]. The annual percentage change (APC) and the average annual percentage change (AAPC) with the corresponding 95% CI were further estimated.

## Results

A total of 177,668 primiparous women were included in this study, of which 175,342 had singleton pregnancies and 2326 had multiparous pregnancies. Table 1 shows the demographic characteristics of nulliparous women, and Table S1 shows the demographic characteristics of all mothers. There were significant differences in demographic characteristics between women undergoing VD and CD. The proportion of VD was higher than that of CD among primipara in 2015–2019 (43.3% vs. 37.1%, *p* < 0.0001). Additionally, more than half of all primiparous women included in this study were aged 25–29 years (60.3%). The proportion of CD among primiparous women aged 30–34 years or > 34 years was significantly higher than that of VD (27.3% vs. 18.8%; 5.3% vs. 1.2%, *p* < 0.0001). The proportion of VD was higher than that of CD among primipara when the GDP per capita was over ¥ 90,000 (25.3% vs. 20.9%; 25.9% vs. 23.9%, *p* < 0.0001). Furthermore, the proportion of pre-term birth among those who underwent CD was higher than that among those who underwent VD (8.9% vs. 5.6%, *p* < 0.0001). The proportion of primiparous women in the first pregnancy accounted for 64.1% of the sample, and primiparous women with more than one pregnancy underwent CD more frequently than they did VD. Additionally, multiparous women reported a higher proportion of CD than that of VD (2.7% vs. 0.2%, *p* < 0.0001). Primiparous women with maternal complications also had a higher proportion of CD (46.1% vs. 21.8%, *p* < 0.0001).

**Table 1:** Characteristics of primipara between 1995 and 2019

Variables	VD	CD	Total	***P***-value
Maternal				
Year				< 0.0001
1995**–**1999	6725 (6.9)	2825 (3.5)	9550 (5.4)	
2000**–**2004	6525 (6.7)	5165 (6.5)	11,690 (6.6)	
2005**–**2009	13,266 (13.6)	15,933 (19.9)	29,199 (16.4)	
2010**–**2014	28,931 (29.6)	26,324 (32.9)	55,255 (31.1)	
2015**–**2019	42,315 (43.3)	29,659 (37.1)	71,974 (40.5)	
Season				< 0.0001
Spring	23,396 (23.9)	19,993 (25.0)	43,389 (24.4)	
Summer	25,272 (25.9)	20,525 (25.7)	45,797 (25.8)	
Autumn	25,774 (26.4)	20,283 (25.4)	46,057 (25.9)	
Winter	23,320 (23.9)	19,105 (23.9)	42,425 (23.9)	
Age (years)				< 0.0001
< 20	429 (0.4)	187 (0.2)	616 (0.4)	
20**–**24	15,747 (16.1)	8587 (10.8)	24,334 (13.7)	
25**–**29	61,980 (63.4)	45,060 (56.4)	107,040 (60.3)	
30**–**34	18,398 (18.8)	21,831 (27.3)	40,229 (22.6)	
> 34	1208 (1.2)	4241 (5.3)	5449 (3.1)	
GDP per capita (¥)				< 0.0001
< 30,000	14,976 (15.3)	9896 (12.4)	24,872 (14.0)	
30,000**–**60,000	15,781 (16.1)	19,079 (23.9)	34,860 (19.6)	
60,000**–**90,000	16,924 (17.3)	15,111 (18.9)	32,035 (18.0)	
90,000**–**120,000	24,722 (25.3)	16,735 (20.9)	41,457 (23.3)	
> 120,000	25,359 (25.9)	19,085 (23.9)	44,444 (25.0)	
Gestational age (weeks)				< 0.0001
< 37	5455 (5.6)	7146 (8.9)	12,601 (7.1)	
37–41	91,826 (93.9)	72,296 (90.5)	164,122 (92.4)	
> 41	481 (0.5)	464 (0.6)	945 (0.5)	
Gravidity				< 0.0001
1	65,608 (67.1)	48,327 (60.5)	113,935 (64.1)	
2–3	29,936 (30.6)	28,185 (35.3)	58,121 (32.7)	
> 3	2218 (2.3)	3394 (4.3)	5612 (3.2)	
Fetus number				< 0.0001
Singleton	97,588 (99.8)	77,754 (97.3)	175,342 (98.7)	
Multiparous	174 (0.2)	2152 (2.7)	2326 (1.3)	
Complications				< 0.0001
Yes	21,355 (21.8)	36,803 (46.1)	58,158 (32.7)	
No/Unclassified	76,407 (78.2)	43,103 (53.9)	119,510 (67.3)	
Neonatal				
Position				< 0.0001
Head	97,075 (99.5)	69,374 (89.2)	166,449 (94.9)	
Breech	202 (0.2)	7723 (9.9)	7925 (4.5)	
Other	21 (0)	226 (0.3)	247 (0.1)	
Unclassified	290 (0.3)	431 (0.6)	721 (0.4)	
Sex				< 0.0001
Male	49,846 (51.1)	41,537 (53.4)	91,383 (52.1)	
Female	47,730 (48.9)	36,212 (46.6)	83,942 (47.9)	
Unclassified	12 (0)	5 (0)	17 (0)	
Weight (g)				< 0.0001
1000**–**1500	327 (0.3)	260 (0.3)	587 (0.3)	
1500**–**2499	3173 (3.3)	3910 (5.0)	7083 (4.0)	
2500**–**3999	90,850 (93.1)	65,328 (84.0)	156,178 (89.1)	
4000**–**4499	3116 (3.2)	7290 (9.4)	10,406 (5.9)	
> 4499	122 (0.1)	966 (1.2)	1088 (0.6)	

Abbreviations: *VD* Vaginal delivery, *CD* Cesarean delivery

For singleton neonates, the head position was the most common (94.9%) (Table 1). Among neonates in the head position, the proportion of VD was higher than that of CD (99.5% vs. 89.2%, *p* < 0.0001). Male newborns had a slightly higher rate of CD than that of VD (53.4% vs. 51.1%, *p* < 0.0001). In addition, newborns with macrosomia also had higher rates of CD (9.4% vs. 3.2%; 1.2% vs. 0.1%, *p* < 0.0001).

**Fig. 1:**
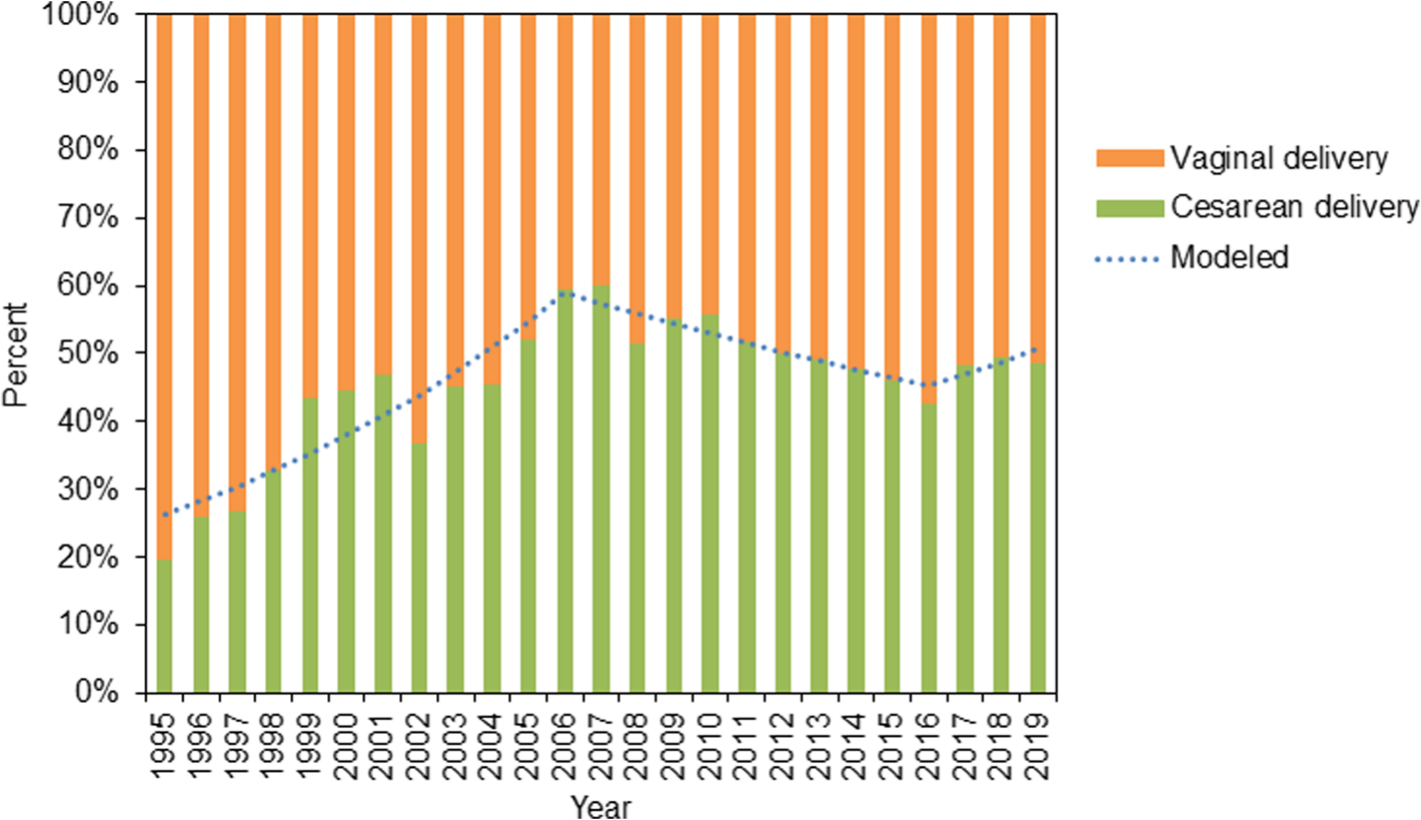
Trends in cesarean delivery rates among primipara during 1995–2019

Figure 1 and Table S2 present the trends among CD rates of primiparous women during 1995–2019. The CD rates showed a significant upward trend during the study period (AAPC: 2.2, 95% CI: 0.6, 3.8%). In 1995–2006, the CD rates continued to increase from 19.1 to 60.5% with an APC of 7.8% (95% CI: 4.8, 10.9%). After 2006, the CD rates started to decline by an APC of − 4.1% (95% CI: − 5.5, − 2.6%). The second inflexion point occurred in 2016. The CD rates showed a non-significant increase from 36.2% in 2016 to 43.2% in 2019 (APC: 3.5, 95% CI: − 3.7, 11.3%).

**Fig. 2:**
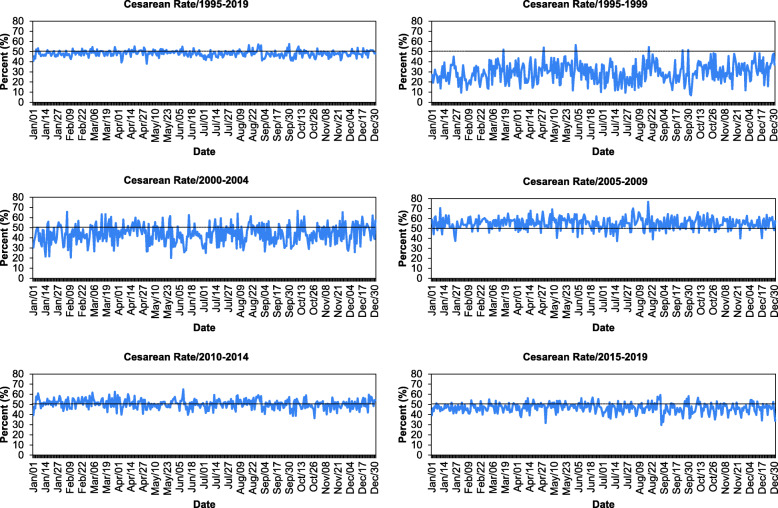
Daily average cesarean delivery rates among primipara during 1995–2019

Figure 2 shows the daily average CD rates among primiparous women during different study periods. In total, the highest CD rate during 1995–2019 was observed on August 28 (57.3%) and the lowest on May 1 (38.7%). With respect to 5–year periods, during 1995–1999, 2000–2004, 2005–2009, 2010–2014, and 2015–2019, the highest CD rates were observed on on June 2 (56.5%), October 9 (66.7%), August 18 (76.7%), June 9 (64.9%), and August 30 (59.24%), respectively. In contrast, the lowest rate was observed on October 3 (6.9%), May 27 (20.0%), July 16 (37.4%), October 27 (37.4%), September 1 (29.7%), respectively.

**Fig. 3:**
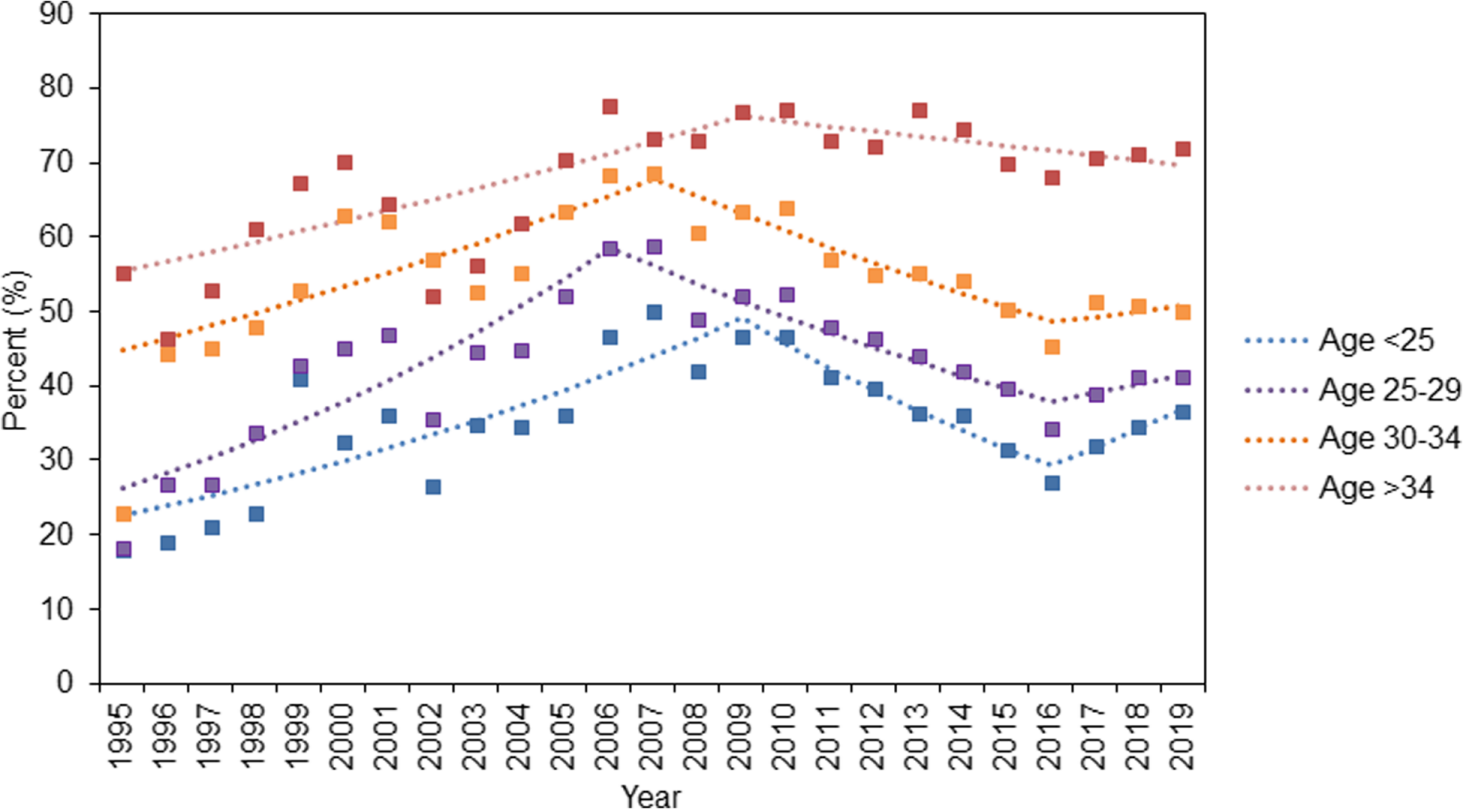
Trends in cesarean delivery rates among primipara stratified by age during 1995–2019

Figure 3 and Table S2 demonstrate the trends in CD rates among primiparous women stratified by age group. Trends in CD rates among primiparous women aged < 25 years showed a significant increase during 1995–2009 (APC: 5.5, 95% CI: 3.2, 7.9%), and then a decrease in 2009–2016 (APC: -7.9, 95% CI: − 12.0, − 3.5%). CD rates among primiparous women aged 25–29 years showed similar trends; they increased with an APC of 7.6% (95% CI: 4.4, 10.8%) in 1995–2006 and decreased with an APC of − 4.9% (95% CI: − 6.5, − 3.3%) in 2006–2016, and then non-significant increases since 2016. CD rates among primiparous women aged 30–34 years and > 34 years showed increasing trends with APC of 3.5% (95% CI: 1.3, 5.8%) and 2.8% (95% CI: 1.0, 4.5%), respectively, followed by decreases in 2007–2019 (APC: -3.6, 95% CI: − 4.9, − 2.4%) and 2005–2019 (APC: -1.4, 95% CI: − 1.7, − 1.1%), respectively.

**Fig. 4:**
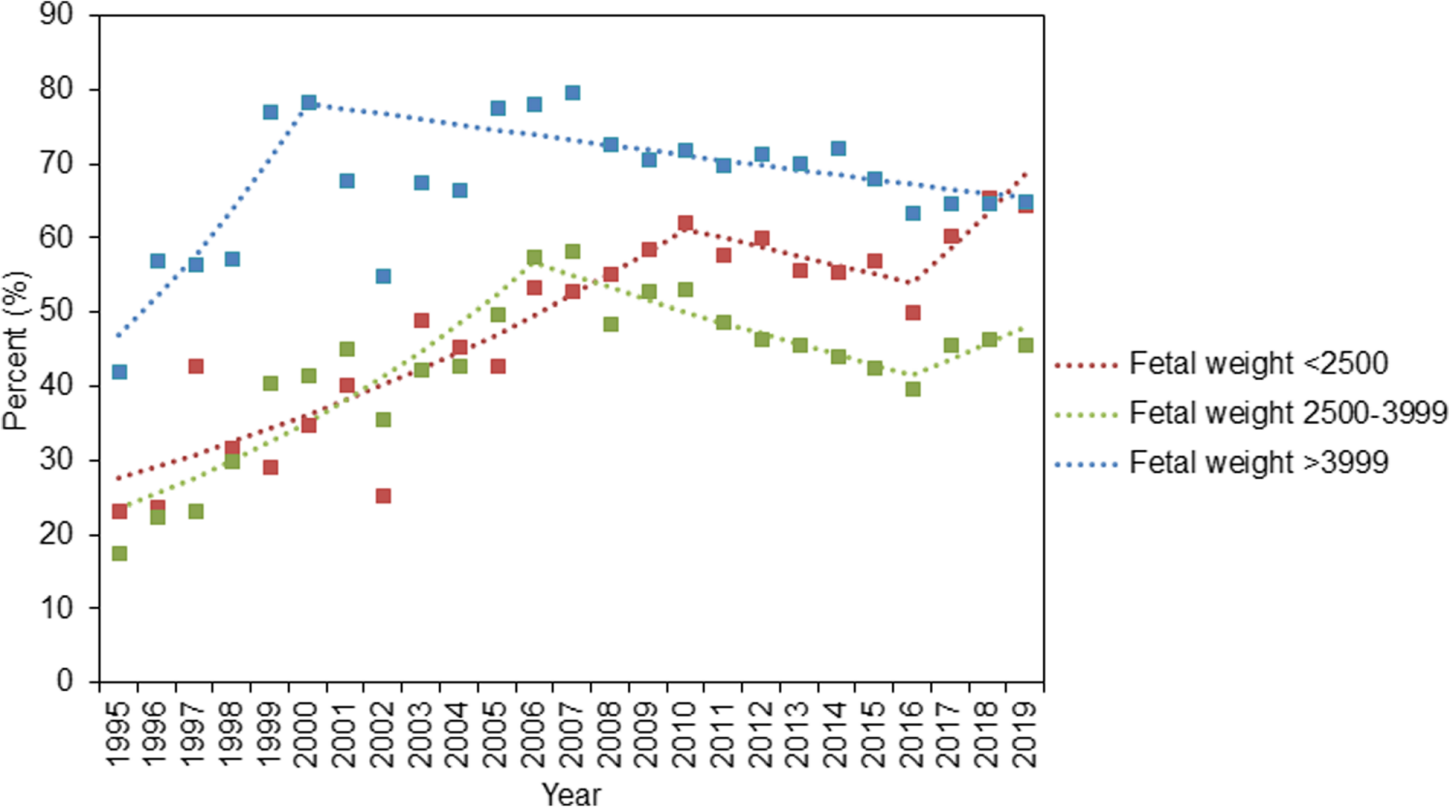
Trends in cesarean delivery rates among primipara with singleton pregnancies stratified by birth weight during 1995–2019

Figure 4 and Table S2 present the trends in CD rates among primiparous women with singleton pregnancies stratified by fetal weight. CD rates among newborns with normal weight demonstrated an upward trend with an APC of 8.6% (95% CI: 5.3, 12.1%) in 1995–2006, and then significantly decreased in 2006–2016 (APC: -4.7, 95% CI: − 6.3, − 3.1%). The trends in CD rates among low-birth-weight newborns increased in 1995–2010 (APC: 5.1, 95% CI: 3.1, 7.2%). After 2016, this trend showed a steeper increase (APC: 14.8, 95% CI: 8.4, 21.7%). Furthermore, the CD rates among those with macrosomia increased in 1995–2000 (APC: 11.3, 95% CI: 1.6, 22.0%) and then slightly decreased in 2000–2019 (APC: -0.9, 95% CI: − 1.4, − 0.3%). Results of multiple sensitivity analysis of trends in CD rates during 2005–2019 are presented in Table S4-S5.

The associations of maternal and neonatal factors with CD among singleton primiparous women are shown in Table 2. Compared with women aged 20–24 years, those with older maternal age (25–29, 30–34, and > 34 years) showed a higher risk of CD in the adjusted model (OR: 1.2, 95% CI: 1.1, 1.3; OR: 1.5, 95% CI: 1.4, 1.6; OR: 1.9, 95% CI: 1.7, 2.2, respectively). Compared with primiparous women with a single pregnancy, those with 2–3 times or > 3 pregnancies had a significantly higher risk of CD (OR: 1.1, 95% CI: 1.0, 1.1; OR: 1.2, 95% CI: 1.1, 1.4, respectively). Furthermore, women giving birth to newborns with low birth weight have a higher risk of CD (OR: 1.2, 95% CI: 1.0, 1.3) and those who gave birth to newborns with macrosomia also showed higher risks of CD (OR: 1.6, 95% CI: 1.5, 1.8; OR: 2.3, 95% CI: 1.7, 3.2, respectively).

**Table 2:** Associations of maternal and neonatal factors with cesarean delivery among primipara with singleton pregnancies

Variables	Unadjusted OR (95% CI)	Adjusted OR (95% CI)
Maternal		
Age (years)		
< 20	0.7 (0.6, 1.0)	0.8 (0.5, 1.2)
20**–**24	Ref	Ref
25**–**29	**1.2 (1.2, 1.3)**	**1.2 (1.1, 1.3)**
30**–**34	**1.7 (1.6, 1.7)**	**1.5 (1.4, 1.6)**
> 34	**2.6 (2.4, 2.7)**	**1.9 (1.7, 2.2)**
Gravidity		
1	Ref	Ref
2–3	**1.1 (1.1, 1.2)**	**1.1 (1.0, 1.1)**
> 3	**1.2 (1.1, 1.3)**	**1.2 (1.1, 1.4)**
Year		
1995**–**1999	Ref	Ref
2000**–**2004	**1.5 (1.4, 1.6)**	**1.4 (1.2, 1.6)**
2005**–**2009	**1.9 (1.8, 2.0)**	**1.3 (1.1, 1.6)**
2010**–**2014	**1.6 (1.5, 1.7)**	**1.3 (1.0, 1.6)**
2015**–**2019	**1.3 (1.3, 1.4)**	1.1 (0.9, 1.4)
Season		
Spring	Ref	Ref
Summer	1.0 (0.9, 1.0)	1.0 (0.9, 1.1)
Autumn	1.0 (0.9, 1.0)	1.0 (0.9, 1.0)
Winter	1.0 (0.9, 1.0)	1.0 (0.9, 1.0)
Neonatal		
Sex		
Male	**1.0 (1.0, 1.1)**	1.0 (1.0, 1.1)
Female	Ref	Ref
Weight (g)		
< 1500	1.0 (0.9, 1.3)	0.9 (0.6, 1.3)
1500**–**2499	**1.4 (1.3, 1.4)**	**1.2 (1.0, 1.3)**
2500**–**3999	Ref	Ref
4000**–**4499	**1.9 (1.8, 2.0)**	**1.6 (1.5, 1.8)**
> 4499	**1.9 (1.8, 2.1)**	**2.3 (1.7, 3.2)**

Adjusted for gestational age, complications, fetal position, and GDP per capita at childbirth year; Bold values represent statistical significance (two-sided *p* < 0.05)

## Discussion

This study reported the trends in CD rates among primiparous women in the past 25 years under the one-child and the two-child policies in China. Overall, the CD rate among primipara in China is much higher than the global average [13]. We found that the CD rates significantly increased from 19.1% in 1995 to 43.2% in 2019, consistent with a previous study conducted among primiparous women from 1993 to 2003 [14]. Furthermore, we found that the CD rates among primipara showed a significant rapid rising trend with an APC of 7.8% during 1995–2006, likely owing to the socioeconomic development in China.

The CD rates among primipara declined significantly from 2006 to 2016. A study conducted by Liao et al. also reported that the CD rates in Hubei Province decreased significantly from 2013 to 2016 [15], and Liu et al. confirmed that the CD rates among primipara with singleton cephalic term pregnancy reduced significantly from 29.3% in 2011 to 16.4% in 2016 [16]. The difference in CD rates between 1995 and 2006 and 2006–2016 are mainly due to the obstetricians’ awareness of CD control and pregnant women’s demands for CD [17].

The elevated risk of maternal and neonatal mortality owing to the high rate of CD had warned health management authorities regarding the need for CD control. In 2001, the sketch of China’s women development (2001–2010), issued by the Health and Family Planning Commission, announced their mission to ‘Reduce unnecessary medical intervention of CD to improve the quality of obstetrics’. After the national policy of CD control was put forward in 2001, it took a few years to formulate and implement effective control measures in various medical institutions. The declining trend during 2006–2016 might be partly explained by the effect of CD control policies [18]. Hospital interventions, including health education for pregnant women and obstetricians on the advantages of VD and the disadvantages of unnecessary CD, might have contributed to the decreasing CD rates [19–21]. Moreover, the introduction of painless delivery and doula care also helped reduce the CD rate [22]. CD control is regarded as health care quality management in obstetric department of the Maternal and Child Health Hospital of Hubei Province, and the use of CD without medical indication was strictly controlled since 2006.

However, this study showed that after the implementation of the two-child policy, the CD rates stopped decreasing after 2016. Researchers have suggested that the increasing trend of CD rates after the implementation of the two-child policy was mainly attributed to the increased proportion of women with previous CD [23]. However, the use of CD among primiparous women under the two-child policy should be taken into consideration.

Interestingly, the highest average daily CD rate in 1995–2019 was observed on August 28, and it changed from June 2 in 1995–1999 to August 30 in 2015–2019. This observation indicates that pregnant women or their families might be likely to choose a good day for CD [8]. Furthermore, the lowest CD rate was observed on September 1 in 2015–2019, consistent with the timing of school reopening in China, which might indicate that many parents use CD instead of VD to prepone the delivery date in order to get their children into school earlier. This conception was particularly obvious in recent years with the rapid socio-economic development of China. It was a great challenge for health managers to control the CD rate caused by social factors [22].

Previous studies have indicated that most women who plan to have a second child were prone to choosing VD for the first child under the two-child policy [8, 24]. Wang et al. conducted in-depth interviews with 45 women, which indicated that most participants were hesitant to have a second child [25]. In this study, the highest proportion of primiparas comprised mothers aged 25–29 years (52.0%). The CD rates in mothers aged < 34 years decreased significantly but showed an inflexion point of increase in 2016. The CD rates among primipara in these age groups did not decrease continuously, which might have been related to the decreased demand of a second child under the two-child policy [26].

Regarding the influence of neonatal characteristics on the risk of CD, this study showed a rapid increase in CD rates in newborns with low birth weight, confirming results from previous studies [27]. Song et al. reported that neonates of low birth weight had a 2-fold higher risk of CD than neonates of normal weight [28], and another study conducted in Taiwan demonstrated an elevated risk of parent-requested CD [29]. For some mothers with pregnancy complications, the use of CD could improve the birth outcome of both mothers and newborns [30]. The increase in the number of primiparous women with advanced age or pregnancy complications might partly explain the rising trend of CD rates among newborns with low birth weight [31]. Moreover, the rapid rising trends in the proportion of newborns with low birth weight might also be attributed to the increased CD rates (Table S3).

This study showed that advanced maternal age at childbirth was positively associated with CD. Mothers with advanced age at childbirth have a higher risk of a wide range of adverse maternal outcomes and neonatal mortality, associated with a more frequent adoption of CD [32–34]. Moreover, mothers with previous miscarriages or abortions have a higher risk of subsequent miscarriage and stillbirth. Consistent with previous studies, the present study also showed an elevated risk of CD among primiparous women with previous miscarriage or abortion history [35, 36].

This study, in line with previous studies, fetal macrosomia was also associated with an elevated risk of CD, which might partly be explained by the elevated risk of stillbirth and birth canal injury during VD in cases of fetal macrosomia [37, 38]. Additionally, the proportion of macrosomia decreased significantly during the whole study period, and the proportion of macrosomia showed a steeper downwards trend during 2016–2019 (Table S3), which might be helpful in long-term CD control.

This study evaluated the progress and challenges of CD control during the social transition in China. However, this study also has some limitations. First, it was based on data from one hospital, and the generalizability of the results to other regions is limited. Second, the data from early years used in this study were registered manually, which may have led to mistakes and omissions. Third, the individual economic and social factors influencing CD rates were not investigated in-depth; therefore, factors influencing CD rates warrant further study. Finally, other fetal factors, including cord around the neck, fetal distress, and asphyxia et al., which are important CD indicators, were excluded from this study, as these data were missing in prior years.

## Conclusions

The overall CD rates among primiparous women increased during 1995–2019. However, the trends in CD rates significantly decreased during the one-child policy period, which might indicate that CD adoption was effectively controlled. However, the CD rates did not continue to decline once the two-child policy was implemented. It has been suggested that interventions including health education, promotion of painless delivery, and doula care could be helpful for CD control. Additionally, social and cultural factors may also contribute to the rising trend in CD rates, which needs to be studied further. The results of this study also suggest that reducing the age at delivery among primipara and controlling fetal macrosomia could decrease the risk of CD.

## Supplementary Information

**Additional file 1:** **Table S1.** Characteristics of maternal mothers between 1995 and 2019. **Table S2.** Joinpoint regression analysis of trends in cesarean delivery rates stratified by age and fetal weight during 1995–2019. **Table S3.** Joinpoint regression analysis of trends in the proportion of fetal weight during 1995–2019. **Table S4.** Joinpoint regression analysis of trends in cesarean delivery rates stratified by age and fetal weight during 2005–2019. **Table S5.** Joinpoint regression analysis of trends in the proportion of fetal weight during 2005–2019.

## Data Availability

Data for this study are available upon reasonable request from the corresponding author with permission from the Ethics Committee of Maternal and Child Health Hospital of Hubei Province.

## Abbreviations

AAPC: Average annual percentage change
APC: Annual percentage change
CD: Cesarean delivery
CI: Confidence interval
OR: Odds ratio
VD: Vaginal delivery
