# Supplementary material for: Trends in cesarean delivery rates in primipara and the associated factors
Source: BMC Pregnancy Childbirth. 2020 Nov 23;20:715. doi: 10.1186/s12884-020-03398-6 (PMC7684937; doi:10.1186/s12884-020-03398-6)
Supplement: Supplementary file 1 — Additional file 1: Table S1. Characteristics of maternal mothers between 1995 and 2019. Table S2. Joinpoint regression analysis of trends in cesarean delivery rates stratified by age and fetal weight during 1995–2019. Table S3. Joinpoint regression analysis of trends in the proportion of fetal weight during 1995–2019. Table S4. Joinpoint regression analysis of trends in cesarean delivery rates stratified by age and fetal weight during 2005–2019. Table S5. Joinpoint regression analysis of trends in the proportion of fetal weight during 2005–2019. [file 12884_2020_3398_MOESM1_ESM.pdf]

Table S1. Characteristics of maternal mothers between 1995 and 2019

Variables	VD	CD	Total	p-value
Maternal				
Year				<0.0001
1995–1999	7527 (6.3)	3177 (2.8)	10704 (4.6)	
2000–2004	7566 (6.3)	5921 (5.3)	13487 (5.8)	
2005–2009	15690 (13.0)	19219 (17.1)	34909 (15.0)	
2010–2014	33568 (27.9)	34411 (30.5)	67979 (29.2)	
2015–2019	55941 (46.5)	50021 (44.4)	105962 (45.5)	
Season				<0.0001
Spring	28531 (23.7)	27714 (24.6)	56245 (24.1)	
Summer	31132 (25.9)	29288 (26.0)	60420 (25.9)	
Autumn	31920 (26.5)	28881 (25.6)	60801 (26.1)	
Winter	28709 (23.9)	26866 (23.8)	55575 (23.9)	
Age (years)				<0.0001
<20	446 (0.4)	205 (0.2)	651 (0.3)	
20–24	16797 (14.0)	9531 (8.5)	26328 (11.3)	
25–29	68773 (57.2)	52408 (46.5)	121181 (52.0)	
30–34	28490 (23.7)	36295 (32.2)	64785 (27.8)	
>34	5786 (4.8)	14310 (12.7)	20096 (8.6)	
Gestational age (weeks)				<0.0001
<37	7194 (6.0)	13073 (11.6)	20267 (8.7)	
37–41	112504 (93.5)	99141 (87.9)	211645 (90.8)	
>41	594 (0.5)	535 (0.5)	1129 (0.5)	
Gravidity				<0.0001
1	65608 (54.5)	48327 (42.9)	113935 (48.9)	
2–3	46731 (38.9)	49786 (44.2)	96517 (41.4)	
>3	7953 (6.6)	14636 (13.0)	22589 (9.7)	
Fetus number				<0.0001
Singleton	119922 (99.7)	108408 (96.2)	228330 (98.0)	
Multiparous	370 (0.3)	4341 (3.9)	4711 (2.0)	
Complications				<0.0001
Yes	26881 (22.4)	55093 (48.9)	81974 (35.2)	
No/Unclassified	93411 (77.7)	57656 (51.1)	151067 (64.8)	
Neonatal				
Position				<0.0001
Head	119221 (99.4)	97726 (90.2)	216947 (95.0)	
Breech	328 (0.3)	9815 (9.1)	10143 (4.4)	
Other	28 (0.0)	354 (0.3)	382 (0.2)	
Unclassified	345 (0.3)	513 (0.5)	858 (0.4)	
Sex				<0.0001
Male	62738 (52.3)	59011 (54.4)	121749 (53.3)	
Female	57170 (47.7)	49391 (45.6)	106561 (46.7)	

Unclassified	14 (0)	6 (0)	20 (0)	
Weight (g)				<0.0001
1000–1500	451 (0.4)	441 (0.4)	892 (0.4)	
1500–2499	4054 (3.4)	5886 (5.4)	9940 (4.4)	
2500–3999	110726 (92.3)	92047 (84.9)	202773 (88.8)	
4000–4499	4466 (3.7)	8844 (8.2)	13310 (5.8)	
>4499	225 (0.2)	1190 (1.1)	1415 (0.6)	

Abbreviations: VD, vaginal delivery; CD, cesarean delivery.

Table S2. Joinpoint regression analysis of trends in cesarean delivery rates stratified by age and fetal weight during 1995–2019

Variables	Trend 1		Trend 2		Trend 3		Full period
	**Period**	**APC**	**Period**	**APC**	**Period**	**APC**	**AAPC**
Total	1995–2006	**7.8 (4.8, 10.9)**	2006–2016	**-4.1 (-5.5, -2.6)**	2016–2019	3.5 (-3.7, 11.3)	**2.2 (0.6, 3.8)**
Age (years)							
<25	1995–2009	**5.5 (3.2, 7.9)**	2009–2016	**-7.9 (-12, -3.5)**	2016–2019	9.2 (-6.9, 28.1)	1.8 (-0.7, 4.5)
25–29	1995–2006	**7.6 (4.4, 10.8)**	2006–2016	**-4.9 (-6.5, -3.3)**	2016–2019	3.7 (-4.8, 13.0)	1.7 (-0.1, 3.5)
30–34	1995–2007	**3.5 (1.3, 5.8)**	2007–2016	**-3.6 (-4.9, -2.4)**	2016–2019	1.5 (-3.4, 6.6)	0.5 (-0.7, 1.8)
>34	1995–2005	**2.8 (1.0, 4.5)**	2005–2019	**-1.4 (-1.7, -1.1)**			0.3 (-0.4, 1.0)
Fetal weight							
Low birth weight	1995–2010	**5.1 (3.1, 7.2)**	2010–2016	-3 (-6.7, 0.8)	2016–2019	**14.8 (8.4, 21.7)**	**4.2 (2.5, 5.8)**
Normal	1995–2006	**8.6 (5.3, 12.1)**	2006–2016	**-4.7 (-6.3, -3.1)**	2016–2019	4.3 (-3.8, 12.9)	**2.3 (0.5, 4.1)**
Macrosomia	1995–2000	**11.3 (1.6, 22.0)**	2000–2019	**-0.9 (-1.4, -0.3)**			1.6 (-0.3, 3.4)

Abbreviations: APC: annual percentage change; AAPC: average annual percentage change; Bold values represent statistical significance (two-sided $p<0.05$)

Table S3. Joinpoint regression analysis of trends in the proportion of fetal weight during 1995–2019

Variables	Trend 1		Trend 2		Trend 3		Full period
	**Period**	**APC**	**Period**	**APC**	**Period**	**APC**	**AAPC**
Low birth weight	1995–2011	**2.8 (0.9, 4.7)**	2011–2016	**-9.9 (-16.7, -2.6)**	2016–2019	**24.5 (12.4, 37.9)**	**2.4 (0.1, 4.7)**
Normal	1995–2004	-0.2 (-0.6, 0.2)	2004–2019	**0.2 (0.1, 0.3)**			0.1 (-0.1, 0.2)
Macrosomia	1995–2016	**-1.2 (-1.8, -0.6)**	2016–2019	**-10.8 (-17.5, -3.7)**			**-2.4 (-3.4, -1.4)**

Abbreviations: APC: annual percentage change; AAPC: average annual percentage change; Bold values represent statistical significance (two-sided $p<0.05$)

Table S4. Joinpoint regression analysis of trends in cesarean delivery rates stratified by age and fetal weight during 2005–2019

Variables	Trend 1		Trend 2		Trend 3		Full period
	Period	APC	Period	APC	Period	APC	AAPC
Total	2005–2016	**-3.9 (-5.0, -2.7)**	2016–2019	3.4 (-3.2, 10.5)			**-2.4 (-3.8, -0.9)**
Age (years)							
<25	2005–2010	1.4 (-4.7, 7.9)	2010–2016	**-8.4 (-13.1, -3.3)**	2016–2019	9.8 (-3.6, 25.0)	-1.2 (-4.6, 2.3)
25–29	2005–2016	**-4.8 (-6.0, -3.6)**	2016–2019	3.6 (-3.6, 11.3)			**-3.0 (-4.6, -1.5)**
30–34	2005–2016	**-3.3 (-4.2, -2.4)**	2016–2019	1.2 (-3.2, 5.8)			**-2.3 (-3.4, -1.3)**
>34	2005–2019	**-1.5 (-1.9, -1.1)**					**-1.5 (-1.9, -1.1)**
Fetal weight							
Low birth weight	2005–2010	5.1 (-1.2, 11.8)	2010–2016	-2.9 (-7.1, 1.4)	2016–2019	**14.6 (6.5, 23.3)**	**3.5 (0.7, 6.3)**
Normal	2005–2016	**-4.5 (-5.8, -3.2)**	2016–2019	4.1 (-3.5, 12.4)			**-2.7 (-4.4, -1.1)**
Macrosomia	2005–2019	**-1.3 (-1.9, -0.8)**					**-1.3 (-1.9, -0.8)**

Abbreviations: APC: annual percentage change; AAPC: average annual percentage change; Bold values represent statistical significance (two-sided $p<0.05$)

Table S5. Joinpoint regression analysis of trends in the proportion of fetal weight during 2005–2019

Variables	Trend 1		Trend 2		Trend 3		Full period
	Period	APC	Period	APC	Period	APC	AAPC
Low birth weight	2005–2013	-0.3 (-4.4, 4.0)	2013–2016	-14.1 (-36.6, 16.3)	2016–2019	**26.5 (11.7, 43.3)**	1.6 (-4.5, 8.1)
Normal	2005–2019	**0.2 (0.1, 0.3)**					**0.2 (0.1, 0.3)**
Macrosomia	2005–2016	-0.7 (-2.0, 0.6)	2016–2019	**-11.3 (-18.2, -3.9)**			**-3.1 (-4.8, -1.3)**

Abbreviations: APC: annual percentage change; AAPC: average annual percentage change; Bold values represent statistical significance (two-sided $p<0.05$)
